# Endoscopic features differentiating non‐*Helicobacter pylori Helicobacter*‐induced gastric mucosa‐associated lymphoid tissue lymphoma with a nodular gastritis‐like appearance and *H. pylori*‐induced conventional nodular gastritis

**DOI:** 10.1111/den.15042

**Published:** 2025-05-09

**Authors:** Yuki Kitadai, Hidehiko Takigawa, Daisuke Shimizu, Misa Ariyoshi, Akiyoshi Tsuboi, Hidenori Tanaka, Ken Yamashita, Yuichi Hiyama, Yoshihiro Kishida, Yuji Urabe, Akira Ishikawa, Toshio Kuwai, Shiro Oka

**Affiliations:** ^1^ Department of Gastroenterology Graduate School of Biomedical and Health Sciences, Hiroshima University Hiroshima Japan; ^2^ Department of Molecular Pathology Graduate School of Biomedical and Health Sciences, Hiroshima University Hiroshima Japan; ^3^ Gastrointestinal Endoscopy and Medicine Hiroshima University Hospital Hiroshima Japan

**Keywords:** gastric MALT lymphoma, *Helicobacter pylori*, NHPH, nodular gastritis, non‐*Helicobacter pylori Helicobacters*

## Abstract

**Objectives:**

Conventional nodular gastritis has been known to be caused by *Helicobacter pylori* infection. Several cases of gastric mucosa‐associated lymphoid tissue (MALT) lymphoma with non‐*H. pylori Helicobacters* (NHPH) exhibit endoscopic findings resembling nodular gastritis. Considering the differences in malignancy, distinguishing between these two conditions is crucial. This study aimed to identify the distinguishing endoscopic features of NHPH‐induced gastric MALT lymphoma with nodular gastritis‐like appearance (NHPHi‐MNG) and *H.*‐induced conventional nodular gastritis (HPi‐NG).

**Methods:**

Between 2011 and 2022, we analyzed 17 patients with NHPHi‐MNG and 50 patients with HPi‐NG at Hiroshima University Hospital and evaluated nodule morphology and distribution patterns.

**Results:**

Compared with the HPi‐NG group, the NHPHi‐MNG group exhibited significantly larger nodules (2.5 vs. 2.0 mm, *P* < 0.05) with protruded morphology (protruded/superficial, elevated: 14/3 vs. 8/42, *P* < 0.05), most prominently in the gastric angulus. The variability in nodule size was significantly higher in the NHPHi‐MNG group than in the HPi‐NG group (0.85 vs. 0.37 mm, *P* < 0.05), reflecting nodule heterogeneity. The distance from the gastric angulus to the proximal end of the nodular lesions was significantly greater in the NHPHi‐MNG group than in the HPi‐NG group (4.4 vs. 1.7 cm, *P* < 0.05). The nodules in the HPi‐NG group were smaller, superficial, elevated, and most prominent in the gastric antrum compared with those in the NHPHi‐MNG group. They were predominantly distributed in the gastric antrum with a homogeneous morphology.

**Conclusion:**

NHPHi‐MNG and HPi‐NG can be endoscopically differentiated according to nodule morphology and distribution. Recognizing these distinguishing features is essential for an accurate diagnosis.

## INTRODUCTION

Nodular gastritis is a specific type of gastritis characterized by a nodular appearance from the gastric antrum to the gastric angulus and is primarily associated with *Helicobacter pylori* (HP) infection.^1^ This condition is more prevalent in children^2^, ^3^, ^4^ and young women^5^, ^6^ than in others and is considered a risk factor for undifferentiated gastric cancer and signet ring cell carcinoma.^7^ Pathologically, nodular gastritis is characterized by hyperplasia and the enlargement of lymphoid follicles with germinal centers in the lamina propria.^2^, ^8^, ^9^, ^10^ Similar endoscopic findings are observed in non‐*H. pylori Helicobacter* (NHPH) gastritis and NHPH‐induced gastric mucosa‐associated lymphoid tissue (MALT) lymphoma, and the pathogenicity of NHPH has recently gained attention.^11^, ^12^, ^13^, ^14^, ^15^

NHPH species are a diverse group of bacteria within the genus *Helicobacter*, excluding HP, and have been identified in humans and animals. Over 50 species of NHPH have been identified, among which *Helicobacter suis* is the most prevalent NHPH species in humans, accounting for 80% of cases,^12^, ^16^, ^17^ followed by *Helicobacter felis*, *Helicobacter bizzozeronii*, *Helicobacter salomonis*, and *Helicobacter heilmannii*. These species are increasingly being recognized for their potential pathogenic roles in diseases.^17^, ^18^, ^19^, ^20^

Gastric MALT lymphoma exhibits various endoscopic patterns,^21^, ^22^ and cases of NHPH‐induced MALT lymphoma presenting with a nodular gastritis‐like appearance have been increasingly reported.^13^, ^14^, ^15^, ^23^ However, the endoscopic differences between NHPH‐induced gastric MALT lymphoma with a nodular gastritis‐like appearance (NHPHi‐MNG) and HP‐induced conventional nodular gastritis (HPi‐NG) have not yet been clarified.

HP infection^24^, ^25^ and the API2‐MALT1 chimeric gene transcript^26^, ^27^, ^28^, ^29^ are widely recognized as causative factors of gastric MALT lymphoma. However, some patients were double‐negative for HP and the *API2*‐*MALT1* transcript. Recent studies have identified NHPH as a potential pathogen in double‐negative cases.^14^, ^30^, ^31^ Additionally, NHPH infection is present in ~55% of patients with gastric MALT lymphoma who are negative for *API2*‐*MALT1* and HP. Furthermore, up to 75% of these patients exhibited a positive response to eradication therapy, suggesting that NHPH infection is a substantial factor influencing treatment response.^14^

Although eradication therapy is a standard treatment and is highly effective for NHPHi‐MNG and HPi‐NG,^32^, ^33^ differences in malignancy between these two conditions may affect the pretreatment evaluation strategy, including the necessity for computed tomography imaging, treatment success rates, and posteradication surveillance strategies. Therefore, it is essential to distinguish between these two conditions before initiating treatment. Furthermore, given the lack of a standardized diagnostic method for NHPH infection and the challenges associated with the histopathological diagnosis of MALT lymphoma, endoscopic findings can provide valuable diagnostic information. Therefore, in this study we focused on identifying the endoscopic features of NHPHi‐MNG and conducted a comparative analysis with those of HPi‐NG.

## METHODS

### Study design and patients

Between 2011 and 2022, 31 patients presenting with nodular gastritis‐like appearance and histologically confirmed MALT lymphoma were identified at Hiroshima University Hospital using esophagogastroduodenoscopy (EGD). We defined the NHPHi‐MNG group as patients who met the following criteria: (i) no mucosal atrophy observed endoscopically; (ii) negative for HP as confirmed using biopsy specimens from five specific regions of the stomach based on the updated Sydney system (USS) – the lesser curvature of the antrum, lesser curvature of the corpus, lesser curvature of the gastric angulus, greater curvature of the antrum, and greater curvature of the corpus; (iii) neutrophil infiltration, atrophy, and intestinal metaplasia graded as 0–1 (regardless of mononuclear cell infiltration); (iv) negative urea breath test (UBT) and stool antigen results; and (v) positive for NHPH infection by polymerase chain reaction (PCR). Seventeen patients met these criteria and were selected for analysis.

For the control group, we extracted 50 cases from 55 patients with a nodular gastritis‐like appearance but without a diagnosis of MALT lymphoma on biopsy. These control cases demonstrated endoscopic evidence of gastric mucosal atrophy and had positive UBT and anti‐HP antibody results, classifying them as HPi‐NG.

Herein, we use “nodular gastritis‐like appearance” to describe the densely distributed nodules observed in NHPHi‐MNG and “nodular gastritis” for conventional HPi‐NG, following our previous report.^13^

**Figure 1:**
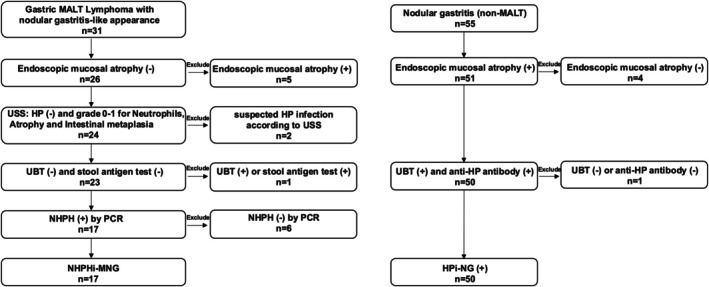
Flowchart of patient selection for non‐*Helicobacter pylori Helicobacter*‐induced gastric mucosa‐associated lymphoid tissue lymphoma with a nodular gastritis‐like appearance (NHPHi‐MNG) and *H. pylori*‐induced conventional nodular gastritis (HPi‐NG). The flowchart illustrates the selection process for patients who underwent esophagogastroduodenoscopy at Hiroshima University Hospital between 2011 and 2022. Seventeen patients with histologically confirmed non‐*H. pylori Helicobacter* (NHPH)‐induced gastric mucosa‐associated lymphoid tissue (MALT) lymphoma presenting with a nodular gastritis‐like appearance and 50 patients with *H. pylori* (HP)‐induced nodular gastritis were included in the analysis. The inclusion criteria for the NHPH‐induced group had negative results for HP infection via serology and polymerase chain reaction (PCR) confirmation of NHPH infection. In the HPi‐NG group, patients demonstrated a positive HP infection with a corresponding nodular gastritis on endoscopy. The exclusion criteria and final distribution of the cases are detailed in the figure. UBT, urea breath test; USS, updated Sydney system.

We compared the clinicopathological features and endoscopic characteristics of the two groups (Fig. 1). All patients underwent follow‐up endoscopy for at least 2 years posttreatment.

### Diagnostic criteria for MALT lymphoma

The diagnosis of MALT lymphoma is based on pathological findings, including lymphoepithelial lesions (LEL) and immunohistochemistry. Immunoglobulin H (*IgH*) gene rearrangement was not used as a definitive diagnostic criterion due to its high false‐positive rate.^34^, ^35^, ^36^, ^37^ The pathological diagnosis was conducted by gastrointestinal pathologists specializing in malignant lymphoma, following the WHO Classification (5th edition).^38^ Particular emphasis was placed on the presence of LEL, with CK AE1/AE3 and CD20 immunostaining used to enhance LEL assessment when necessary. Additionally, follicular colonization, where lymphoma cells infiltrate pre‐existing lymphoid follicles, was a key distinguishing feature of gastric MALT lymphoma. To differentiate follicular colonization from reactive lymphoid follicular hyperplasia in gastritis, immunostaining for CD10, Bcl‐2, and Ki‐67 was performed to assess polarity‐associated staining pattern changes.

### Evaluation methods for HP and NHPH status

NHPH infection was diagnosed by excluding participants with UBT or stool antigen positivity to rule out HP coinfection. This approach was based on previous studies reporting that all NHPH cases were negative for HP stool antigen tests and the possibility of false‐positive UBT results in NHPH infection.^11^ For HP diagnosis, UBT positivity was required in addition to serological antibody testing to ensure the exclusion of NHPH coinfection, considering the potential for false‐positive serological results in NHPH cases.^11^, ^39^

### Pathological assessment of background mucosa based on USS

We incorporated background mucosal assessment based on the USS to exclude cases of coinfection with HP and NHPH. In HP‐negative gastric mucosa, all parameters of the USS are classified as Grade 0. While generally milder than in HP infections, parameters of the USS in NHPH infections may still exhibit slight neutrophil infiltration, mucosal atrophy, and particularly pronounced mononuclear cell infiltration.^40^ Therefore, to definitively exclude HP‐infection cases from the NHPH infection group, we required an HP score of 0. For neutrophil infiltration, atrophy, and intestinal metaplasia scores, we allowed cases with scores up to Grade 1, while mononuclear cell scores were not restricted.

### Evaluation of endoscopic characteristics

Endoscopic images from EGD were retrospectively evaluated by three board‐certified gastrointestinal endoscopists with over 10 years of experience, confirming that all patients were in a pre‐eradication state.

Endoscopic assessments focused on the lesion extent and nodule morphology. The lesion extent was evaluated based on the distance from the gastric angulus to the most proximal nodular lesion. The size of the nodules was defined as the maximum horizontal diameter. Regarding nodule height, we followed the internationally accepted Paris classification,^41^ defining nodules ≥2.5 mm as the protruded type and those <2.5 mm as the superficial‐elevated type. Size irregularities were assessed using the standard deviation. Gastric mucosal atrophy was evaluated using the Kimura–Takemoto classification.^42^ NHPH has a lower affinity for the gastric epithelium than HP,^11^, ^43^ and it induces less mucosal atrophy.^40^, ^44^, ^45^ Therefore, an absence of gastric mucosal atrophy was used as a diagnostic criterion to distinguish NHPH from HP infections. Finally, we evaluated the diagnostic accuracy of NHPHi‐MNG using endoscopic features as predictive factors.

### DNA extraction and gastric *Helicobacter* species‐specific PCR assay

DNA was extracted from tissue samples obtained from the antrum, angulus, and corpus for pathological diagnosis using an AllPrep DNA/RNA Kit (Qiagen Japan, Tokyo, Japan). DNA concentration was measured using a NanoDrop spectrophotometer (Thermo Fisher Scientific, Waltham, MA, USA). PCR amplification of the urease gene from the NHPH species (*H. suis*, *H. bizzozeronii*, *H. felis*, *H. salomonis*, and *H. heilmannii*) was conducted with buffer and DNA polymerase from KOD FX Neo (Toyobo, Osaka, Japan), following the manufacturer's instructions. The settings and primers used for the PCR were based on those used in previously reported protocols.^14^

HP and NHPH are distinguished by morphology and size in hematoxylin and eosin staining,^46^ with Giemsa staining also used at our institution. However, detection remains challenging due to low bacterial load and uneven distribution, reducing diagnostic sensitivity.^23^, ^47^ Thus, we adopted PCR as the gold standard for NHPH detection.

### Statistical analysis

Differences between groups were assessed using the Mann–Whitney *U‐*test for quantitative data and the *χ* ^2^‐test for categorical variables. Fisher's exact test was performed as required. All statistical analyses were conducted using EZR software (Saitama Medical Center, Jichi Medical University, Saitama, Japan). All tests were two‐sided, with a *P*‐value of <0.05 considered statistically significant.^48^

## RESULTS

### Patient characteristics

This study included 17 patients with NHPHi‐MNG and 50 patients with HPi‐NG.

In the NHPHi‐MNG group, eight patients (47.1%) were female and nine (52.9%) were male, with a mean age of 48 years (range: 24–76 years). In all patients, *H. suis* was identified as the bacterial species. Complete remission (CR) of MALT lymphoma was achieved following one or two courses of eradication therapy, with resolution of nodular lesions observed in all patients. Molecular analysis revealed that the *IgH* gene rearrangement was present in 14 patients, whereas the *API2*‐*MALT1* chimeric gene transcript was not detected in any patient.

In the HPi‐NG group, 37 patients (74%) were female and 13 (26%) were male, with a mean age of 41 years (range: 12–77 years). Gastric mucosal atrophy was classified as the closed type in 32 patients and the open type in 18 patients. In all 44 patients in whom successful HP eradication was confirmed, endoscopy revealed the disappearance of nodular gastritis. In contrast, nodular gastritis persisted in all six patients in whom HP eradication was unsuccessful. Detailed patient characteristics are shown in Table 1.

**Table 1:** Patient characteristics in the non‐*Helicobacter pylori Helicobacter*‐induced gastric mucosa‐associated lymphoid tissue lymphoma with a nodular gastritis‐like appearance (NHPHi‐MNG) and *H. pylori*‐induced conventional nodular gastritis (HPi‐NG) groups

	NHPHi‐MNG	HPi‐NG
	*n* = 17	*n* = 50
Age, years (range)	48 (24–76)	41 (12–77)
Sex		
Female	8 (47%)	37 (74%)
Male	9 (53%)	13 (26%)
Number of HP eradication treatments		
Once	15 (88%)	42 (84%)
Twice	2 (12%)	8 (16%)
Disappearance of nodules		
Yes (after successful eradication treatment)	17 (100%)	44 (88%)
No (after successful eradication treatment)	0 (0%)	0 (0%)
No (after unsuccessful eradication treatment)	0 (0%)	6 (12%)
Mucosal atrophy		
None	17 (100%)	0 (0%)
Closed‐type	0 (0%)	32 (64%)
Open‐type	0 (0%)	18 (36%)
Species of *Helicobacter*		
HP	0 (0%)	50 (100%)
*H. suis*	17 (100%)	0 (0%)
Other NHPHs	0 (0%)	0 (0%)
*IgH* gene rearrangement		
Positive	14 (82%)	3 (6%)
Negative	3 (18%)	47 (94%)
*API2*‐*MALT1* chimeric gene transcript		
Positive	0 (0%)	–
Negative	17 (100%)	–

HP, *H. pylori*; NHPH, non‐*H. pylori Helicobacter*; Other NHPHs, *H. suis*, *H. bizzozeronii*, *H. felis*, *H. salomonis*, and *H. heilmannii*s.

### Comparison of clinicopathological features

**Figure 2:**
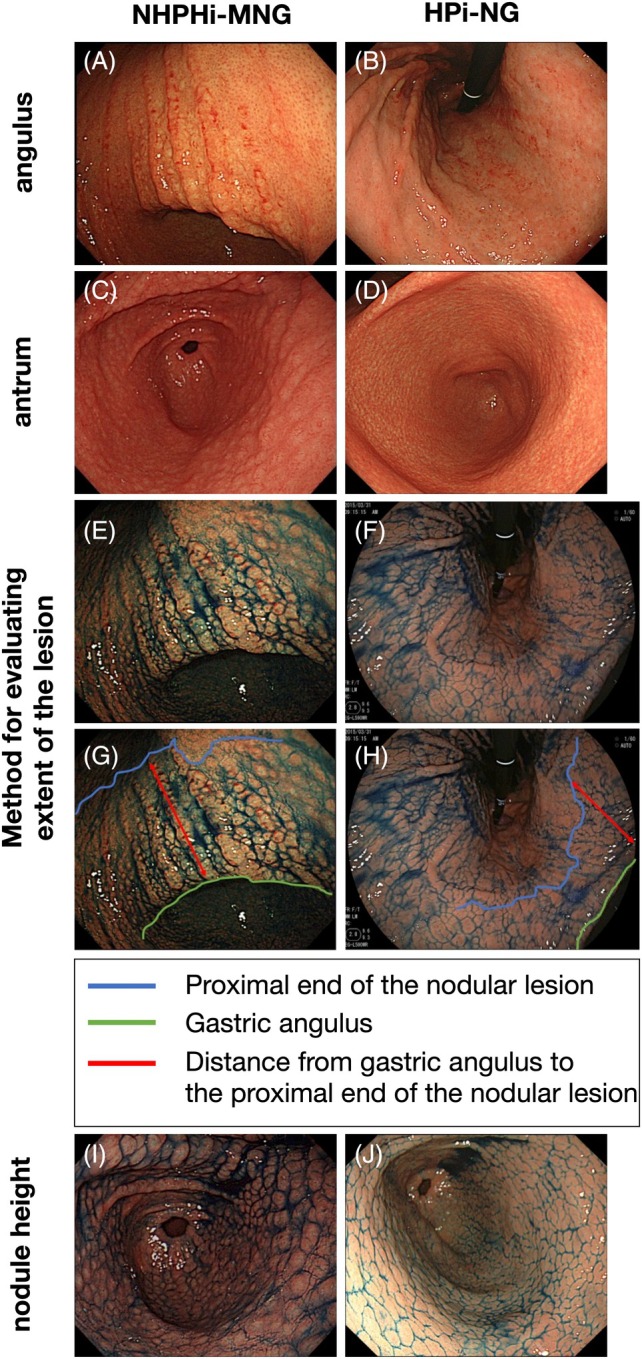
Comparison of distribution patterns of nodules. (A) Multiple heterogeneous, coarse nodules extend beyond the gastric angulus into the lower corpus in the non‐*Helicobacter pylori Helicobacter*‐induced gastric mucosa‐associated lymphoid tissue lymphoma with a nodular gastritis‐like appearance (NHPHi‐MNG) group, whereas (B) no nodules were observed in the corpus region in the *H. pylori*‐induced conventional nodular gastritis (HPi‐NG) group. (C) Nodular lesions are most pronounced at the gastric angulus in the HPi‐NG group compared with the antrum in the NHPHi‐MNG group. (D) HPi‐NG exhibits finely distributed nodules concentrated primarily in the antrum. (E–H) The distance from the gastric angulus to the most proximal nodular lesion is indicated in the images. Lesion extent in the NHPHi‐MNG group is notably broader than that in the HPi‐NG group, often extending beyond the gastric angulus and into the lower corpus of the stomach. (I, J) Indigo carmine highlights nodules more clearly in the (I) NHPHi‐MNG group than in the (J) HPi‐NG group.

**Figure 3:**
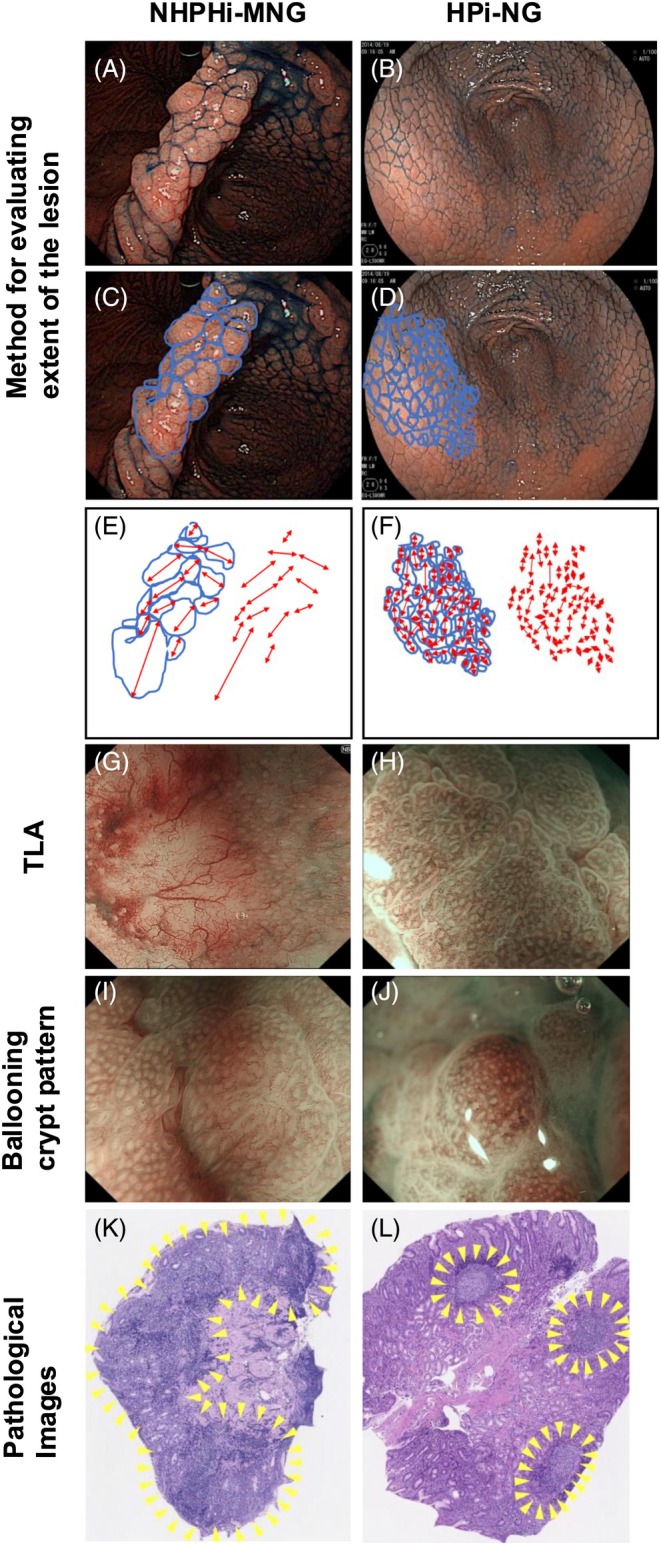
Comparison of variability of nodules and narrow‐band imaging (NBI) findings. Each nodule was traced on endoscopic images, with the maximum diameter measured and an evaluation of size variability conducted. (A–F) Nodules in the non‐*Helicobacter pylori Helicobacter*‐induced gastric mucosa‐associated lymphoid tissue lymphoma with a nodular gastritis‐like appearance (NHPHi‐MNG) group are significantly larger and display greater heterogeneity in elevation than those in the *H. pylori*‐induced conventional nodular gastritis (HPi‐NG) group. (G, H) Observations from magnified endoscopy combined with NBI reveal the disappearance of gland structure and branching abnormal blood vessels in the NHPHi‐MNG group (G); such findings are absent in the HPi‐NG group (H). (I, J) Both groups show a ballooning crypt pattern. (K, L) Comparison of pathological findings. In the NHPHi‐MNG group, prominent nodules were frequently observed, often merging with adjacent nodules. In contrast, the HPi‐NG group showed uniform lymphoid follicles with preserved polarity. TLA, tree‐like appearance.

HPi‐NG was predominantly observed in female patients, whereas NHPHi‐MNG was more frequently diagnosed in male patients (female/male: 8/9 vs. 37/13, *P* = 0.07). Endoscopic assessments focused on the lesion extent and nodule morphology. Representative endoscopic images of each disease are shown in Figures 2 and 3, and quantitative comparisons and statistical analyses of the endoscopic features are presented in Table 2. Compared with lesions in the HPi‐NG group, those in the NHPHi‐MNG group were notably broader, often extending beyond the gastric angulus and into the lower corpus of the stomach (antrum alone/beyond the angulus: 0/17 vs. 18/32, *P* < 0.05; Fig. 2A,B). Nodular lesions were most intense at the gastric angulus in the NHPHi‐MNG group, whereas they were uniformly distributed in the antrum in the HPi‐NG group (antrum/angulus: 6/11 vs. 49/1, *P* < 0.05; Fig. 2C,D). A comparative analysis of the proximal spread, based on the distance between the most proximal nodule and the gastric angulus, indicated a significantly greater proximal spread in the NHPHi‐MNG group than in the HPi‐NG group (4.4 ± 2.1 cm vs. 1.7 ± 1.6 cm, *P* < 0.05; Fig. 2E–H). Regarding nodule morphology, the NHPHi‐MNG group exhibited a significantly higher frequency of protruded‐type lesions than the HPi‐NG group (protruded/superficial, elevated: 14/3 vs. 8/42, *P* < 0.05; Fig. 2I,J). A size comparison showed that the nodules in the NHPHi‐MNG group were significantly larger than those in the HPi‐NG group (2.5 mm vs. 2.0 mm, *P* < 0.05). Additionally, heterogeneity in nodule size, assessed by standard deviation, was significantly greater in the NHPHi‐MNG group than in the HPi‐NG group (0.85 ± 0.25 mm vs. 0.37 ± 0.14 mm, *P* < 0.05; Fig. 3A–F). Based on narrow‐band imaging (NBI) endoscopic observation, a tree‐like appearance (TLA) at the nodule apex was consistently found only in NHPHi‐MNG cases and was absent in all HPi‐NG cases (TLA (+)/(−): 7/10 vs. 0/15, *P* < 0.05; Fig. 3G,H). Finally, in the comparison of pathological findings, prominent nodule formation was observed in the NHPHi‐MNG group, with adjacent nodules often found to be fused together. In contrast, uniform lymphoid follicles with preserved polarity were observed in the HPi‐NG group (Fig. 3K,L).

**Table 2:** Comparison of characteristics between the NHPHi‐MNG and HPi‐NG groups

	NHPHi‐MNG	HPi‐NG	*P*‐value
	*n* = 17	*n* = 50	
Age, years (range)	48 (24–76)	41 (12–77)	0.1
Sex			
Female	8 (47%)	37 (74%)	0.07
Male	9 (53%)	13 (26%)	
Disappearance of nodules after eradication therapy (excluding eradication failure cases)			
Yes	17 (100%)	44 (100%)	1.0
No	0 (0%)	0 (0%)	
Location (spreading of nodules)			
Antrum alone	0 (0%)	18 (36%)	0.003
Beyond the angulus	17 (100%)	32 (64%)	
Lesions where nodular gastritis‐like changes are most intense			
Antrum	6 (35%)	49 (98%)	<0.001
Angulus	11 (65%)	1 (2%)	
Distance from the gastric angulus to the proximal end of the nodular lesion, ±SE (cm)	4.4 ± 2.1	1.7 ± 1.6	<0.001
Morphology of the nodules			
Superficial, elevated	3 (18%)	42 (84%)	<0.001
Protruded	14 (82%)	8 (16%)	
Maximum size of the nodules, ± SE (mm)	2.5 ± 0.5	2.0 ± 0.3	<0.001
Average standard deviation of nodule sizes	0.85 ± 0.25	0.37 ± 0.14	<0.001
TLA (excluding unknown cases)			
(+)	7 (41%)	0 (0%)	0.008
(−)	10 (59%)	15 (100%)	

SE, standard error; TLA, tree‐like appearance.

### Diagnostic accuracy of endoscopic features to distinguish NHPHi‐MNG from HPi‐NG

**Figure 4:**
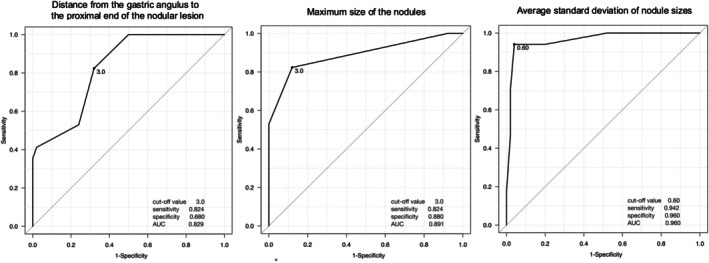
Receiver operating characteristic (ROC) curve analysis for determining the optimal cut‐off values for continuous variables. The optimal cut‐off value for the distance from the gastric angulus to the proximal end of the nodular lesion is 3 cm (area under the ROC curve [AUC] 0.822, sensitivity 0.824, specificity 0.680, accuracy 0.716). The maximum nodule size has a cut‐off value of 3 mm (AUC 0.859, sensitivity 0.824, specificity 0.880, accuracy 0.866). The cut‐off value for the average standard deviation of nodule sizes is 0.6 mm (AUC 0.971, sensitivity 0.941, specificity 0.960, accuracy 0.955).

We evaluated the sensitivity, specificity, and diagnostic accuracy of endoscopic findings for NHPHi‐MNG. According to receiver operating characteristic curve analysis, sensitivity/specificity/accuracy based on the distance from the gastric angulus to the proximal end of the nodular lesion, maximum nodule size, and average standard deviation of nodule sizes were 0.824/0.680/0.716, 0.824/0.880/0.866, and 0.941/0.960/0.955, respectively (Fig. 4).

For categorical variables, sensitivity/specificity/accuracy based on nodules beyond the angulus, lesions with the most intense nodular gastritis‐like changes at the angulus, nodule morphology (protruded type), and the presence of TLA (+) were 1.000/0.360/0.522, 0.647/0.980/0.896, 0.882/0.800/0.821, and 0.412/1.000/0.688, respectively (Table 3).

**Table 3:** Endoscopic features for non‐*Helicobacter pylori Helicobacter*‐induced gastric mucosa‐associated lymphoid tissue lymphoma with a nodular gastritis‐like appearance: Sensitivity, specificity, and accuracy rates

Endoscopic features	Sensitivity	Specificity	Accuracy rate
Distance from the gastric angulus to the proximal end of the nodular lesion (≥3 cm)	0.824	0.680	0.716
Maximum size of the nodules (≥3 mm)	0.824	0.880	0.866
Average standard deviation of nodule sizes (≥0.6 mm)	0.941	0.960	0.955
Location (beyond the angulus)	1.000	0.360	0.522
Lesions where nodular gastritis‐like changes are most intense (angulus)	0.647	0.980	0.896
Morphology of the nodules (protruded)	0.882	0.800	0.821
TLA (+)	0.412	1.000	0.688

non‐*Helicobacter pylori Helicobacter*‐induced gastric mucosa‐associated lymphoid tissue lymphoma with a nodular gastritis‐like appearance; TLA, tree‐like appearance.

Diagnostic performance was also assessed based on the number of endoscopic findings. When endoscopic features were used as diagnostic predictors, the accuracy reached a maximum of 0.965 when four or more factors were present (Table 4).

**Table 4:** Sensitivity, specificity, and accuracy based on the number of endoscopic predictive factors for non‐*Helicobacter pylori Helicobacter*‐induced gastric mucosa‐associated lymphoid tissue lymphoma with a nodular gastritis‐like appearance

Number of endoscopic predictive factors	Sensitivity	Specificity	Accuracy rate
1 or more	1.000	0.300	0.478
2 or more	1.000	0.600	0.701
3 or more	1.000	0.840	0.881
4 or more	1.000	0.950	0.965
5 or more	0.824	1.000	0.955

## DISCUSSION

Here we clarified the endoscopic characteristics of NHPHi‐MNG, with HPi‐NG as the comparison. Compared with HPi‐NG, NHPHi‐MNG exhibited larger, variably sized, and coarser nodules of greater height, which were densely distributed from the antrum to the lower corpus of the stomach. The most prominent nodules were consistently observed in the lesser curvature of the gastric angulus.

Our analysis revealed significant differences in nodule morphology and distribution between the groups, highlighting variations in nodule size and the extent of involvement. Nodule size differences likely stem from tumorigenesis rather than inherent distinctions between HP and NHPH infections. The contrast between reactive lymphoid hyperplasia and neoplastic proliferation in MALT lymphoma explains this variation. Compared with HP‐associated nodular gastritis, where hyperplasia remains reactive, monoclonal proliferation in MALT lymphoma leads to large nodules. Additionally, lymphomatous nodules may merge with adjacent nodules, forming irregular structures, explaining the coarse, heterogeneous endoscopic appearance.^49^ Regarding nodular extent, HP infection distributes evenly, inducing uniform antral mucosal changes via localized immune responses. In contrast, NHPH forms infection foci mainly at gland borders, resulting in uneven distribution.

The TLA and ballooning crypt patterns are also well‐known findings on NBI‐magnifying endoscopy of gastric MALT lymphoma.^50^, ^51^ In this study, ballooning crypt patterns were observed in NHPHi‐MNG and HPi‐NG cases, suggesting that these findings reflect compressive changes due to hyperplasia of lymphoid follicles within the lamina propria, rather than being specific to gastric MALT lymphoma (Fig. 3I,J). However, TLA was exclusively observed in NHPHi‐MNG cases.

We calculated diagnostic accuracy based on the number of predictive factors and found it reached 96.5% with four or more factors (Table 4). However, as endoscopic findings alone cannot ensure 100% accuracy, combining them with PCR or histopathology is crucial when feasible. Yet, PCR remains challenging in current clinical practice, and while histopathology is common, its sensitivity is limited.^23^, ^47^

New diagnostic methods have been increasingly reported in recent years, including *H. suis* culture from the gastric mucosa^52^ and serological enzyme‐linked immunosorbent assay (ELISA) for *H. suis* infection, offer promise for a more accessible diagnosis. Notably, ELISA‐based diagnostics for *H. suis* have been reported, demonstrating a sensitivity of 100% and a specificity of 92.5% for *H. suis* detection, which could be one of the main reliable tests for NHPH infection.^53^

As previously reported, HPi‐NG shows a high rate of improvement after successful eradication therapy; in this study, all cases of successful HP eradication resulted in complete regression of HP‐induced nodular gastritis.^54^, ^55^, ^56^ For NHPHi‐MNG, all patients demonstrated nodule regression, achieving CR of MALT lymphoma. Nakamura *et al*. reported the eradication success rate of NHPH using antibiotic therapy (45/45 cases), highlighting the high effectiveness of this approach.^17^ In previous studies of *API2*‐*MALT1*‐negative, HP‐negative, and NHPH‐positive gastric MALT lymphomas, a CR rate of 75% was achieved following eradication therapy.^16^ Here we observed a 100% success rate for NHPH eradication and CR in the NHPHi‐MNG cases. These results suggest that NHPH is a key pathogen in gastric MALT lymphoma and support the efficacy of antibiotic treatment.

This study examined NHPH infections in cases with nodule formation; however, some NHPH infections do not lead to nodules. Our previous study found that 81% (13/16) of NHPH‐positive gastric MALT lymphoma cases had no nodules, including some *H. suis*‐positive cases.^14^ Additionally, Nakamura *et al*. analyzed 236 cases of gastric diseases, identifying 49 NHPH‐positive cases, of which 34 (69%) had no nodule formation.^17^ In this study, all NHPHi‐MNG cases were associated with *H. suis* infection, suggesting a potential link between *H. suis* infection and nodule formation. However, as some *H. suis*‐infected cases did not develop nodules, the pathological mechanisms remain unclear. Further research is needed to investigate the mechanisms of nodule formation in NHPH‐associated gastric MALT lymphoma.

We focused on differentiating NHPHi‐MNG from HPi‐NG, but benign nodular lesions in NHPH gastritis also exist,^11^, ^12^, ^15^ with smaller nodule variations making a distinction from HPi‐NG more challenging and an ongoing issue. Several endoscopic features of NHPH gastritis have been reported, including nodular gastritis,^11^ the white marbled appearance,^47^ crack‐like mucosa,^12^ and spotty redness.^15^ However, Okamura *et al*.^40^ found no significant difference in the prevalence of the white marbled appearance or crack‐like mucosa between HP‐associated and NHPH gastritis, instead identifying the absence of diffuse redness as a key distinction. Tsukadaira *et al*.^12^ and Kadota *et al*.^15^ reported that regular arrangement of collecting venules in the gastric body differentiates NHPH gastritis from HP infection. Takeda *et al*.^57^ found that linked color imaging enhances the visibility of nodular gastritis, crack‐like mucosa, white marbled appearance, erosions, and uneven redness, suggesting that advances in image‐enhanced endoscopy aid NHPH gastritis diagnosis.

This study has some limitations. It was a retrospective cohort analysis performed in a single hospital. Additionally, the rarity of NHPH infections limited the sample size. The nucleic acid extraction samples used for PCR were part of clinical biopsy specimens, and due to the nature of these specimens, it was difficult to retrospectively add new cases or incorporate cases from other institutions. Therefore, further multicenter studies with larger cohorts are needed to validate these findings.

In conclusion, NHPHi‐MNG and HPi‐NG can be endoscopically differentiated according to nodule morphology and distribution. Recognizing these distinguishing features is essential for an accurate diagnosis.

## CONFLICT OF INTEREST

Authors declare no conflict of interest for this article.

## FUNDING INFORMATION

None.

## ETHICS STATEMENT

Approval of the research protocol by an Institutional Reviewer Board: This study was approved by the Institutional Review Board of Hiroshima University Hospital (approval number: E‐298) and conducted in accordance with the tenets of the Declaration of Helsinki.

Informed consent: The need for informed consent was waived owing to the use of anonymized data. However, we used an opt‐out method for participation in this study.

Registry and the Registration No. of the study/trial: N/A.

Animal studies: N/A.

## Data Availability

The data supporting the findings of this study are available from the corresponding author upon request.

## References

[1] Shiotani A , Kamada T , Kumamoto M *et al*. Nodular gastritis in Japanese young adults: Endoscopic and histological observations. J Gastroenterol 2007; 42: 610–615. 17701123 10.1007/s00535-007-2073-5

[2] Bahú G , da Silveira TR , Maguilnick I , Ulbrich‐Kulczynski J . Endoscopic nodular gastritis: An endoscopic indicator of high‐grade bacterial colonization and severe gastritis in children with *Helicobacter pylori* . J Pediatr Gastroenterol Nutr 2003; 36: 217–222. 12548057 10.1097/00005176-200302000-00011

[3] Mazigh Mrad S , Abidi K , Brini I , Boukthir S , Sammoud A . Nodular gastritis: An endoscopic indicator of Helicobacter pylori infection in children. Tunis Med 2012; 90: 789–792. 23197056

[4] Rafeey M , Jafari Rouhi AH , Gassemi BA , Rouhi AJ . Relationship between endoscopic nodular gastritis and *Helicobacter pylori* infection in children. Indian J Gastroenterol 2004; 23: 138–139. 15333969

[5] Miyamoto M , Haruma K , Yoshihara M *et al*. Nodular gastritis in adults is caused by *Helicobacter pylori* infection. Dig Dis Sci 2003; 48: 968–975. 12772798 10.1023/a:1023016000096

[6] Nakamura S , Mitsunaga A , Imai R *et al*. Clinical evaluation of nodular gastritis in adults. Dig Endosc 2007; 19: 74–79.

[7] Choi HJ , Lee SY , Lee JH *et al*. Two atypical cases of nodular gastritis: A poorly differentiated gastric adenocarcinoma and a pseudo‐low grade gastric MALT lymphoma. Gastroenterol Res 2010; 3: 41–45. 10.4021/gr2010.02.170w PMC5139839 27956984

[8] Nakashima R , Nagata N , Watanabe K *et al*. Histological features of nodular gastritis and its endoscopic classification. J Dig Dis 2011; 12: 436–442. 22118692 10.1111/j.1751-2980.2011.00532.x

[9] Okamura T , Sakai Y , Hoshino H , Iwaya Y , Tanaka E , Kobayashi M . Superficially located enlarged lymphoid follicles characterise nodular gastritis. Pathology 2015; 47: 38–44. 25474513 10.1097/PAT.0000000000000195

[10] Nagata T , Ishitake H , Shimamoto F *et al*. Histopathological study of the relationship between lymphoid follicles and different endoscopic types of nodular gastritis. Rinsho Byori 2014; 62: 1031–1039. Japanese. 27509717

[11] Goji S , Tamura Y , Sasaki M *et al*. *Helicobacter suis*‐infected nodular gastritis and a review of diagnostic sensitivity for *Helicobacter heilmannii*‐like organisms. Case Rep Gastroenterol 2015; 9: 179–187. 26120299 10.1159/000431169 PMC4478311

[12] Tsukadaira T , Hayashi S , Ota H *et al*. Prevalence, clinical features, and esophagogastroduodenoscopy (EGD) findings of non‐*Helicobacter pylori Helicobacter* infection: A study of 50 cases at a single facility in Japan. Helicobacter 2021; 26: e12811. 33908121 10.1111/hel.12811

[13] Takigawa H , Masaki S , Naito T *et al*. Helicobacter suis infection is associated with nodular gastritis‐like appearance of gastric mucosa‐associated lymphoid tissue lymphoma. Cancer Med 2019; 8: 4370–4379. 31210418 10.1002/cam4.2314 PMC6675707

[14] Takigawa H , Yuge R , Masaki S *et al*. Involvement of non‐*Helicobacter pylori* helicobacter infections in *Helicobacter pylori*‐negative gastric MALT lymphoma pathogenesis and efficacy of eradication therapy. Gastric Cancer 2021; 24: 937–945. 33638751 10.1007/s10120-021-01172-x

[15] Kadota H , Yuge R , Miyamoto R *et al*. Investigation of endoscopic findings in nine cases of *Helicobacter suis*‐infected gastritis complicated by gastric mucosa‐associated lymphoid tissue lymphoma. Helicobacter 2022; 27: e12887. 35363918 10.1111/hel.12887

[16] Haesebrouck F , Pasmans F , Flahou B *et al*. Gastric helicobacters in domestic animals and nonhuman primates and their significance for human health. Clin Microbiol Rev 2009; 22: 202–223 Table of Contents. 19366912 10.1128/CMR.00041-08 PMC2668234

[17] Nakamura M , Øverby A , Michimae H *et al*. PCR analysis and specific immunohistochemistry revealing a high prevalence of non‐*Helicobacter pylori* helicobacters in *Helicobacter pylori*‐negative gastric disease patients in Japan: High susceptibility to an HP eradication regimen. Helicobacter 2020; 25: e12700. 32790220 10.1111/hel.12700

[18] Liu J , He L , Haesebrouck F *et al*. Prevalence of coinfection with gastric non‐*Helicobacter pylori Helicobacter* (NHPH) species in *Helicobacter pylori*‐infected patients suffering from gastric disease in Beijing, China. Helicobacter 2015; 20 (4): 284–290. 25510739 10.1111/hel.12201

[19] Øverby A , Murayama SY , Michimae H *et al*. Prevalence of gastric non‐*Helicobacter pylori*‐helicobacters in Japanese patients with gastric disease. Digestion 2017; 95 (1): 61–66. 28052279 10.1159/000452400

[20] Bahadori A , De Witte C , Agin M *et al*. Presence of gastric *Helicobacter* species in children suffering from gastric disorders in southern Turkey. Helicobacter 2018; 23 (5): e12511. 29974550 10.1111/hel.12511

[21] Kelessis NG , Vassilopoulos PP , Tsamakidis KG , Bai MG , Avital S , Rosenthal RJ . Is gastroscopy still a valid diagnostic tool in detecting gastric MALT lymphomas? A dilemma beyond the eye. Mucosa‐associated lymphoid tissue. Surg Endosc 2003; 17: 469–474. 12404054 10.1007/s00464-002-8544-0

[22] Montalbán C , Castrillo JM , Abraira V *et al*. Gastric B‐cell mucosa‐associated lymphoid tissue (MALT) lymphoma. Clinicopathological study and evaluation of the prognostic factors in 143 patients. Ann Oncol 1995; 6 (4): 355–362. 7619750 10.1093/oxfordjournals.annonc.a059184

[23] Okiyama Y , Matsuzawa K , Hidaka E , Sano K , Akamatsu T , Ota H . *Helicobacter heilmannii* infection: Clinical, endoscopic and histopathological features in Japanese patients. Pathol Int 2005; 55: 398–404. 15982214 10.1111/j.1440-1827.2005.01844.x

[24] Wündisch T , Thiede C , Morgner A *et al*. Long‐term follow‐up of gastric MALT lymphoma after *Helicobacter pylori* eradication. J Clin Oncol 2005; 23 (31): 8018–8024. 16204012 10.1200/JCO.2005.02.3903

[25] Moleiro J , Ferreira S , Lage P , Dias Pereira A . Gastric MALT lymphoma: Analysis of a series of consecutive patients over 20 years. U Eur Gastroenterol J 2016; 4 (3): 395–402. 10.1177/2050640615612934 PMC4924435 27403306

[26] Sagaert X , De Wolf‐Peeters C , Noels H , Baens M . The pathogenesis of MALT lymphomas: Where do we stand? Leukemia 2007; 21: 389–396. 17230229 10.1038/sj.leu.2404517

[27] Nakamura S , Ye H , Bacon CM *et al*. Clinical impact of genetic aberrations in gastric MALT lymphoma: A comprehensive analysis using interphase fluorescence in situ hybridisation. Gut 2007; 56: 1358–1363. 17525089 10.1136/gut.2007.123729 PMC2000261

[28] Nakamura S , Matsumoto T , Nakamura S *et al*. Chromosomal translocation t(11;18)(q21;q21) in gastrointestinal mucosa associated lymphoid tissue lymphoma. J Clin Pathol 2003; 56: 36–42. 12499431 10.1136/jcp.56.1.36 PMC1769850

[29] Ye H , Liu H , Raderer M . High incidence of t(11;18)(q21;q21) in *Helicobacter pylori*‐negative gastric MALT lymphoma. Blood 2003; 101 (7): 2547–2550. 12517817 10.1182/blood-2002-10-3167

[30] Asano N , Iijima K , Koike T , Imatani A , Shimosegawa T . *Helicobacter pylori*‐negative gastric mucosa‐associated lymphoid tissue lymphomas: A review. World J Gastroenterol 2015; 21: 8014–8020. 26185372 10.3748/wjg.v21.i26.8014 PMC4499343

[31] Morgner A , Lehn N , Andersen LP *et al*. *Helicobacter heilmannii*‐associated primary gastric low‐grade MALT lymphoma: Complete remission after curing the infection. Gastroenterology 2000; 118: 821–828. 10784580 10.1016/s0016-5085(00)70167-3

[32] Nakamura S , Hojo M . Diagnosis and treatment for gastric mucosa‐associated lymphoid tissue (MALT) lymphoma. J Clin Med 2022; 12: 120. 36614921 10.3390/jcm12010120 PMC9820981

[33] Zucca E , Bertoni F . The spectrum of MALT lymphoma at different sites: Biological and therapeutic relevance. Blood 2016; 127: 2082–2092. 26989205 10.1182/blood-2015-12-624304

[34] Fend F , Schwaiger A , Weyrer K *et al*. Early diagnosis of gastric lymphoma: Gene rearrangement analysis of endoscopic biopsy samples. Leukemia 1994; 8: 35–39. 8289496

[35] Akahane M , Uehara T , Hidaka E *et al*. Detection of monoclonality of gastric MALT lymphoma using PCR method and its clinicopathological application. J Clin Pathol 2003; 51: 852–858. 14560652

[36] Zhang GP , Cao PF , Feng LJ . Detection and clinical significance of genes in primary gastrointestinal MALT lymphoma. Tumour Biol 2014; 35: 3223–3228. 24272086 10.1007/s13277-013-1421-8

[37] Hsi ED , Greenson JK , Singleton TP , Siddiqui J , Schnitzer B , Ross CW . Detection of immunoglobulin heavy chain gene rearrangement by polymerase chain reaction in chronic active gastritis associated with *Helicobacter pylori* . Hum Pathol 1996; 27: 290–296. 8600045 10.1016/s0046-8177(96)90071-4

[38] Nagtegaal ID , Odze RD , Klimstra D *et al*. The 2019 WHO classification of tumours of the digestive system. Histopathology 2020; 76: 182–188. 31433515 10.1111/his.13975 PMC7003895

[39] Jhala D , Jhala N , Lechago J , Haber M . *Helicobacter heilmannii* gastritis: Association with acid peptic diseases and comparison with *Helicobacter pylori* gastritis. Mod Pathol 1999; 12: 534–538. 10349993

[40] Okamura T , Iwaya Y , Nagaya T *et al*. Diagnosis by combination of endoscopic findings helps differentiate non‐*Helicobacter pylori Helicobacter*‐infected gastritis from *Helicobacter pylori*‐infected gastritis. Helicobacter 2024; 29: e13070. 38514917 10.1111/hel.13070

[41] Participants in the Paris Workshop . The Paris endoscopic classification of superficial neoplastic lesions: Esophagus, stomach, and colon. Gastrointest Endosc 2003; 58 (Supplement): S3–S43. 14652541 10.1016/s0016-5107(03)02159-x

[42] Kimura K , Takemoto T . An endoscopic recognition of the atrophic border and its significance in chronic gastritis. Endoscopy 1969; 1: 87–97.

[43] Debongnie JC , Donnay M , Mairesse J . *Gastrospirillum hominis* (“*Helicobacter heilmanii*”): A cause of gastritis, sometimes transient, better diagnosed by touch cytology? Am J Gastroenterol 1995; 90: 411–416. 7872280

[44] Ierardi E , Monno RA , Gentile A *et al*. *Helicobacter heilmannii* gastritis: A histological and immunohistochemical trait. J Clin Pathol 2001; 54: 774–777. 11577125 10.1136/jcp.54.10.774 PMC1731280

[45] Joo M , Kwak JE , Chang SH *et al*. *Helicobacter heilmannii*‐associated gastritis: Clinicopathologic findings and comparison with *Helicobacter pylori*‐associated gastritis. J Korean Med Sci 2007; 22: 63–69. 17297253 10.3346/jkms.2007.22.1.63 PMC2693570

[46] Kobayashi M , Yamamoto K , Ogiwara N , Matsumoto T , Shigeto S , Ota H . *Helicobacter heilmannii*‐like organism in parietal cells: A diagnostic pitfall. Pathol Int 2016; 66: 120–122. 26345479 10.1111/pin.12349

[47] Shiratori S , Mabe K , Yoshii S *et al*. Two cases of chronic gastritis with non‐*Helicobacter pylori Helicobacter* infection. Intern Med 2016; 55: 1865–1869. 27432094 10.2169/internalmedicine.55.5891

[48] Kanda Y . Investigation of the freely available easy‐to‐use software ‘EZR’ for medical statistics. Bone Marrow Transplant 2013; 48: 452–458. 23208313 10.1038/bmt.2012.244 PMC3590441

[49] Wotherspoon AC , Doglioni C , Isaacson PG . Low‐grade gastric B‐cell lymphoma of mucosa‐associated lymphoid tissue (MALT): A multifocal disease. Histopathology 1992; 20: 29–34. 1737623 10.1111/j.1365-2559.1992.tb00912.x

[50] Nonaka K , Ishikawa K , Arai S *et al*. A case of gastric mucosa‐associated lymphoid tissue lymphoma in which magnified endoscopy with narrow band imaging was useful in the diagnosis. World J Gastrointest Endosc 2012; 4: 151–156. 22523617 10.4253/wjge.v4.i4.151 PMC3329616

[51] Hassegawa RT , Ogawa EKM , El Ibrahim R , Venco FE , Maruta LM . Pre‐malignant signs of gastric MALT lymphoma. Autops Case Rep 2020; 10: e2019130. 32039061 10.4322/acr.2019.130 PMC6945307

[52] Rimbara E , Suzuki M , Matsui H *et al*. Isolation and characterization of *Helicobacter suis* from human stomach. Proc Natl Acad Sci USA 2021; 118: e2026337118. 33753513 10.1073/pnas.2026337118 PMC8020762

[53] Matsui H , Rimbara E , Suzuki M *et al*. Development of serological assays to identify *Helicobacter suis* and *H. pylori* infections. iScience 2023; 26 (4): 106522. 37123222 10.1016/j.isci.2023.106522 PMC10139984

[54] Ze D . Analysis of the relationship between Helicobacter pylori and nodular gastritis in children. Chin J Clin Gastroenterol 2009. Available from: https://www.oriprobe.com/journals/lcxhbzz/2009_6.html

[55] Nishizawa T , Sakitani K , Suzuki H *et al*. Clinical features of cardiac nodularity‐like appearance induced by *Helicobacter pylori* infection. World J Gastroenterol 2020; 26: 5354–5361. 32994693 10.3748/wjg.v26.i35.5354 PMC7504245

[56] Chen MJ , Shih SC , Wang TE , Chan YJ , Chen CJ , Chang WH . Endoscopic patterns and histopathological features after eradication therapy in *Helicobacter pylori*‐associated nodular gastritis. Dig Dis Sci 2008; 53: 1893–1897. 18080192 10.1007/s10620-007-0097-6

[57] Takeda T , Asaoka D , Hirai S , Nakamura M , Nagahara A . Endoscopic features with linked‐color imaging of non‐*Helicobacter pylori Helicobacter*‐associated gastritis: A case study. VideoGIE 2024; 9: 123–127. 38482475 10.1016/j.vgie.2023.10.009 PMC10927495

